# Simvastatin, but not pravastatin, inhibits the proliferation of esophageal adenocarcinoma and squamous cell carcinoma cells: a cell-molecular study

**DOI:** 10.1186/s12944-018-0946-7

**Published:** 2018-12-22

**Authors:** Yan Chen, Li-Bin Li, Jun Zhang, Du-Peng Tang, Jing-Jing Wei, Ze-Hao Zhuang

**Affiliations:** 1 Department of Endoscopy, The First Affiliated Hospital of Fujian Medical University, Fuzhou, Fujian 350000 People’s Republic of China; 2 Department of Gastroenterology, The Second Affiliated Hospital of Fujian Medical University, Quanzhou, Fujian 362000 People’s Republic of China; 3 Department of Gastroenterology, The People’s Hospital Affiliated to Fujian University Of Traditional Chinese Medicine, Fuzhou, Fujian 350000 People’s Republic of China

**Keywords:** Statin, Esophageal squamous carcinoma cell, Esophageal adenocarcinoma cell, Proliferation

## Abstract

**Background and objective:**

Long-term statin therapy has been shown to protect against several cancers, including esophageal cancer (EC). While the mechanisms underlying this effect are not clear. We investigated the effect of hydrophobic simvastatin and hydrophilic pravastatin on the proliferation of EC cells and sought to explore the underlying mechanisms.

**Methods:**

Esophageal adenocarcinoma OE-19 cells and esophageal squamous cell carcinoma Eca-109 cells were treated with different concentrations of simvastatin or pravastatin for 24 h and 48 h. Cell proliferation was assessed by Cell Counting Kit-8 assay. Malondialdehyde (MDA) levels were measured by thiobarbituric acid (TBA) assay. mRNA and protein expression of COX-2 were determined by reverse transcriptase-polymerase chain reaction (RT-PCR) and Western blot, respectively; The expression of prostaglandin E_2_ (PGE_2_) was measured by ELISA.

**Results:**

Simvastatin, but not pravastatin, significantly inhibited the proliferation of OE-19 and Eca-109 cells in a dose- and time-dependent manner, accompanying with the increasing of the MDA level. Moreover, simvastatin suppressed the expression of COX-2 and PGE_2_ in both OE-19 and Eca-109 cells in a dose-dependent manner.

**Conclusions:**

Lipophilic simvastatin, but not hydrophilic pravastatin, had significant inhibitory effects on the proliferation of Eca-109 and OE-19 cells. The reduction of COX-2 and PGE_2_ by simvastatin suggested that the inhibitory effect of simvastatin on the proliferation of EC cells may be independent of its lipid-lowering effect. Simvastatin may be a promising agent for the prevention and treatment of EC.

## Background

Esophageal adenocarcinoma (EAC) and esophageal squamous cell carcinoma (ESCC) are two different pathological types of esophageal cancer (EC). The worldwide incidence of both EAC and ESCC are still high, however, there is a lack of effective agents for the prevention and treatment of EC.

Statins are human hydroxymethylglutaryl coenzymeA (HMG-CoA) reductase inhibitors that include both lipophilic (lovastatin and simvastatin) and hydrophilic (pravastatin and rosuvastatin) agents, which are widely used for the treatment of hypercholesterolemia and prevention of cardiovascular diseases [1]. In the past several decades, experimental evidence has shown that statins could potentially play a role in preventing EC. The mechanisms proposed include upregulation of pro-apoptotic proteins such Bad and Bax, as well as reduced cell proliferation and enhanced apoptosis through reduced activation of signalling G-proteins as a consequence of reduced levels of melavonate due to inhibition of the HMG-CoA reductase [2, 3]. Several clinical studies have shown that statins can reduce cancer risk significantly [4, 5]. However, the underlying molecular mechanisms are not clear.

A meta-analysis showed that stains were associated with reduced risk of EC [6]. However, the reported data were not disaggregated by histopathological subtype of EC. In a case-control study [7], statins were shown to reducing the risk of EAC by 48%, but there is no study investigating the role of statins in the prevention of ESCC. Since ESCC is the dominant type of EC in China, it should be interesting to study the effects of statins on the prevention of ESCC.

Different types of statins may show different chemopreventive effects. Ogunwobo [8] has found that lipophilic simvastatin, but not hydrophilic pravastatin, can inhibit proliferation of EAC FLO-1 cells in a dose-dependent manner. An epidemiological study [9] found that both the two types of statins demonstrated negative associations with the development of EAC (OR = 0.58; 95% CI: 0.39–0.87), esophagogastric junctional adenocarcinoma (OR = 0.29; 95% CI: 0.09–0.92) and ESCC (OR = 0.51; 95% CI: 0.27–0.98). However, it is still not unclear whether the mechanism of the two types of statins protecting cells from EC is different.

Downregulation of cyclooxygenase-2 (COX-2) is closely associated with the occurrence and development of EC. It was found that statins inhibit expression of COX-2, regardless of in vitro or in vivo study [10]. However, differences between the inhibitory effects of lipophilic and hydrophilic statins are not well-characterized.

In this study, we investigated the effect of lipophilic simvastatin and hydrophilic pravastatin on the proliferation of esophageal cancer adenocarcinoma OE-19 cells and esophageal squamous cell carcinoma Eca-109 cells, and analyzed their effect on the expression of COX-2 and its downstream product PGE2.

## Materials and methods

### Cell lines and reagents

EAC OE-19 cells and ESCC Eca-109 cells were purchased from Shanghai Bioleaf Biotech (Shanghai, China). Simvastatin and pravastatin were purchased from Sigma (Poole, United Kindom). Cell Counting Kits-8 (CCK-8) were purchased from Beyotime (Shanghai, China). PGE_2_ metabolite ELISA Kits were purchased from Colorful Gene (Wuhan, China). Thiobarbituric acid (TBA) assay kits were purchased from Nanjing Jiancheng Bioengineering Institute (Nanjing, China).

### Cell culture and group dividing

EAC OE-19 cells and ESCC Eca-109 cells were grown in RPMI 1640 (GibcoBRL, Grand Island, NY, USA) supplemented with 100 units/mL penicillin G, 100 μg/mL streptomycin and 10% fetal bovine serum (GibcoBRL) in an incubator at 37 °C, 95% humidity, and with 5% carbon dioxide. The cells were treated with various concentrations of simvastatin or pravastatin (15 μM, 30 μM, 45 μM, 60 μM and 75 μM) for 24 and 48 h. Control cells were treated with equal amount of dimethylsulfoxide (DMSO). Each concentration did five parallel, repeated 3 times.

### Cell proliferation

4 × 10^3^ cells per well were subcultured in 96-well plates in 10% FCS-containing medium for 72 h. After 24 h incubation, the cells were treated with different concentrations of simvastatin or pravastatin for 24, 48 and 72 h. At the indicated time points, 10 μL CCK-8 was added to each well and cultured for 4 h. Upon removal of the supernatant, 150 μL DMSO was added to dissolve formazan. The optical density 450 nm value was measured by enzyme-linked immunoabsorbent assay (ELISA). Survival rates were calculated from the absorbance values.

### Malondialdehyde

Malondialdehyde (MDA) content was determined by thiobarbituric acid (TBA) assay kit according to the manufacturer’s instructions. 4 × 10^3^/well were subcultured in 96-well plates for 24 h. Cells were then treated with cell lysis buffer and phenylmethyl sulfonylfluoride. MDA was calculated according to the following formula: cell supernatant MDA (nmol/mg) = (Experiment group OD- blank control OD)/(Standard group OD- blank control OD) × Standard concentration (10 nmol/mL)/Sample concentration (mg/mL). Each group was repeated in triplicate.

### RT-PCR

Total RNA was isolated using Trizol reagent (Life Technologies, Gaithersburg, MD, USA) in culture flasks and EP tubes. For reverse transcriptase (RT)-PCR, cDNA was synthesized by the Superscript II First Strand Synthesis System (Life Technologies, Gaithersburg, MD, USA). The cDNA was amplified by PCR using primers specific for COX-2 (sense: 5’-CAA AAGCTGGGAAGCCTTCT-3′: antisense: 5′- CCATCC TTGAAAAGGCGCAG-3′) and GADPH (as control) (sense: 5’-GGGTGTGAAC CATGAGAAGT-3′; antisense: 5′- GGCATGGACTGTGGTCATGA-3′). Prelimina- ry experiments were conducted to ensure that the PCR conditions were at the logarithmic phase of the reaction for each set of primers. PCR conditions were: 94 °C for 5 min followed by 94 °C for 30 s, 59 °C for 30 s, 72 °C for 60 s (forty cycles), and a final extension step at 72 °C for 5 min. PCR products were run on 2% agarose gels and analyzed using a gel documentation system (Ultra-Violet Product Limited, CA, USA). The relative expression levels were determined by comparing the density to the controls.

### Western blot

Whole-cell lysates were prepared by resuspending cell pellets in lysis buffer [20 mM Tris–HCl (pH 7.4), 150 mM NaCl, 1 mM EDTA, 1 mM EGTA, 1% Triton X-100, 2.5 mM sodium pyrophosphate, 1 mM β-glycerophosphate, 1 mM Na3VO4, 1 μg/mL leupeptin, 1 μg/mL aprotinin, 1 μM PMSF] in culture flasks. Protein concentrations were measured using DC Protein Assay kit (Bio-Rad, Hercules, CA). Equal amount of proteins (30 μg) were separated with 10% SDS–polyacrylamide gel and transferred on to a PVDF membrane (Amersham, Piscataway, NJ). Membranes were blocked with 5% *w*/*v* non-fat dry milk dissolved in Tris buffered saline with Tween-20 (TBS-T; 0.1% Tween-20; pH 8.3) at room temperature for 1 h, then incubated with primary antibodies at 4 °C overnight. The primary antibodies used were rabbit COX-2 or β-actin antibodies (Santa Cruz, CA). After washing with TBS-T, membranes were incubated with horseradish peroxidase (HRP)-labeled secondary antibodies (Sigma, USA) for 30–45 min at room temperature. Immunobands were visualized using enhanced chemiluminescence (ECL) kit (Amersham). The amounts of each protein was quantified relative to that of β-actin by densitometry.

### PGE_2_ levels

PGE_2_ levels were detected by ELISA. The detection followed the manufacturer’s instructions.

### Statistical analysis

SPSS 16.0 software was used for analysis of data. Quantitative data were represented as mean ± standard deviation. Multi-group comparison was done using one-way ANOVA and multiple comparisons among the groups were performed by least-significant difference (LSD). Using analysis of Kruskal-Wallis H to compare the group differences, when variance uneven using Mann-Whitney U. Inspection level was 0.05, *P* < 0.05 was considered statistically significant.

## Results

### Simvastatin, but not pravastatin, inhibited the proliferation of EC cells

**Fig. 1:**
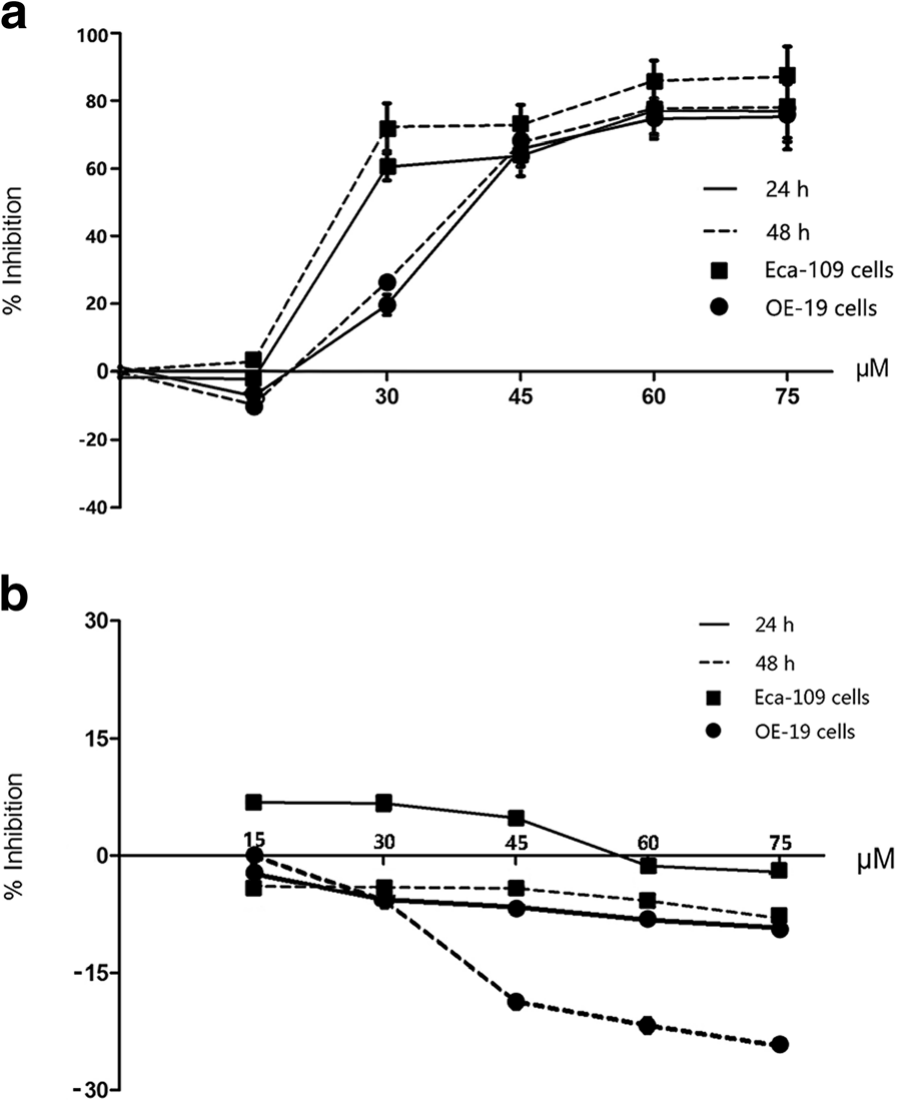
Proliferation of Eca-109 cells and OE-19 cells after simvastatin or pravastatin treatment. OE-19 and Eca-109 cells were treated with 0.1% DMSO or different doses (15 μM, 30 μM, 45 μM, 60 μM and 75 μM) of Simvastatin (**a**) or pravastatin (**b**) for 24 and 48 h. Cell viability was assessed with CCK-8 assay. *Eca-109, Esophageal squamous cell carcinoma; OE-19, esophageal adenocarcinoma; DMSO, dimethylsulfoxide*

Both OE-19 and Eca-109 cell proliferation was blunted by simvastatin in a dose- and time- dependant manner, with statin concentrations of 30 μM and above producing statistically significant reductions (Fig. 1a). In contrast, there was no obvious difference in the effects of pravastatin did on the proliferation of OE-19 or Eca-109 cells (*P* > 0.05) (Fig. 1b).

### Simvastatin, but not pravastatin, up-regulated the level of MDA

**Fig. 2:**
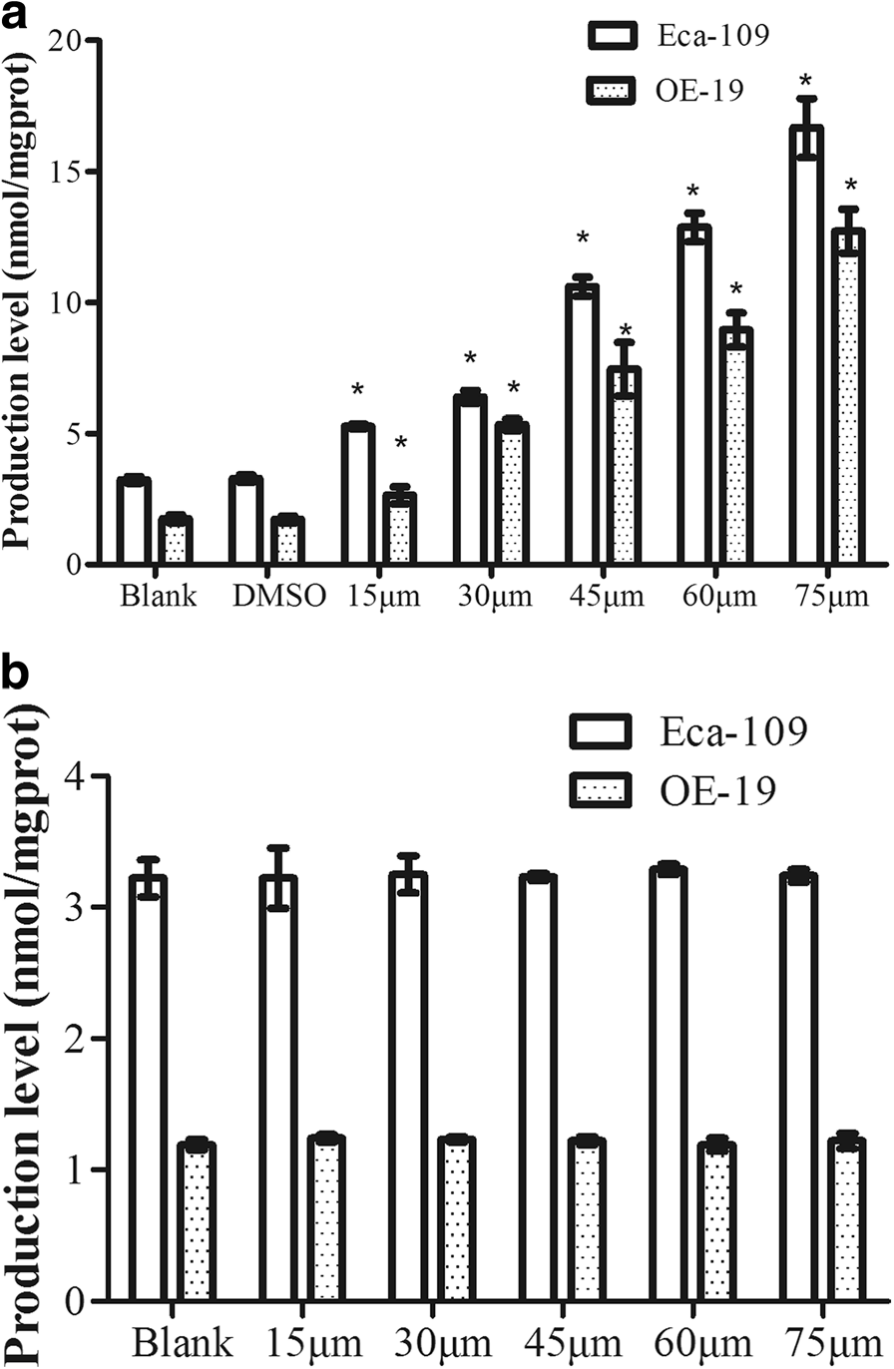
MDA production in Eca-109 and OE-19 cells treated with simvastatin or pravastatin. OE-19 and Eca-109 cells were treated with 0.1% DMSO or different doses (15 μM, 30 μM, 45 μM, 60 μM and 75 μM) of Simvastatin (**a**) or pravastatin (**b**) for 24 h. MDA levels were determined using TBA assay. * indicates significant difference from DMSO group (*P* < 0.05). *MDA, Malondialdehyde; Eca-109, Esophageal squamous cell carcinoma; OE-19, esophageal adenocarcinoma*; DMSO, *dimethylsulfoxide*; TBA, *thiobarbituric acid*

Compared to the blank and control groups, simvastatin groups significantly increased the level of MDA in a dose-dependent manner (*P* < 0.05) (Fig. 2a). However, the promoting of pravastatin on the level of MDA in either of EC cells was not great (*P* > 0.05) (Fig. 2b).

### Simvastatin inhibited the expression of COX-2

**Fig. 3:**
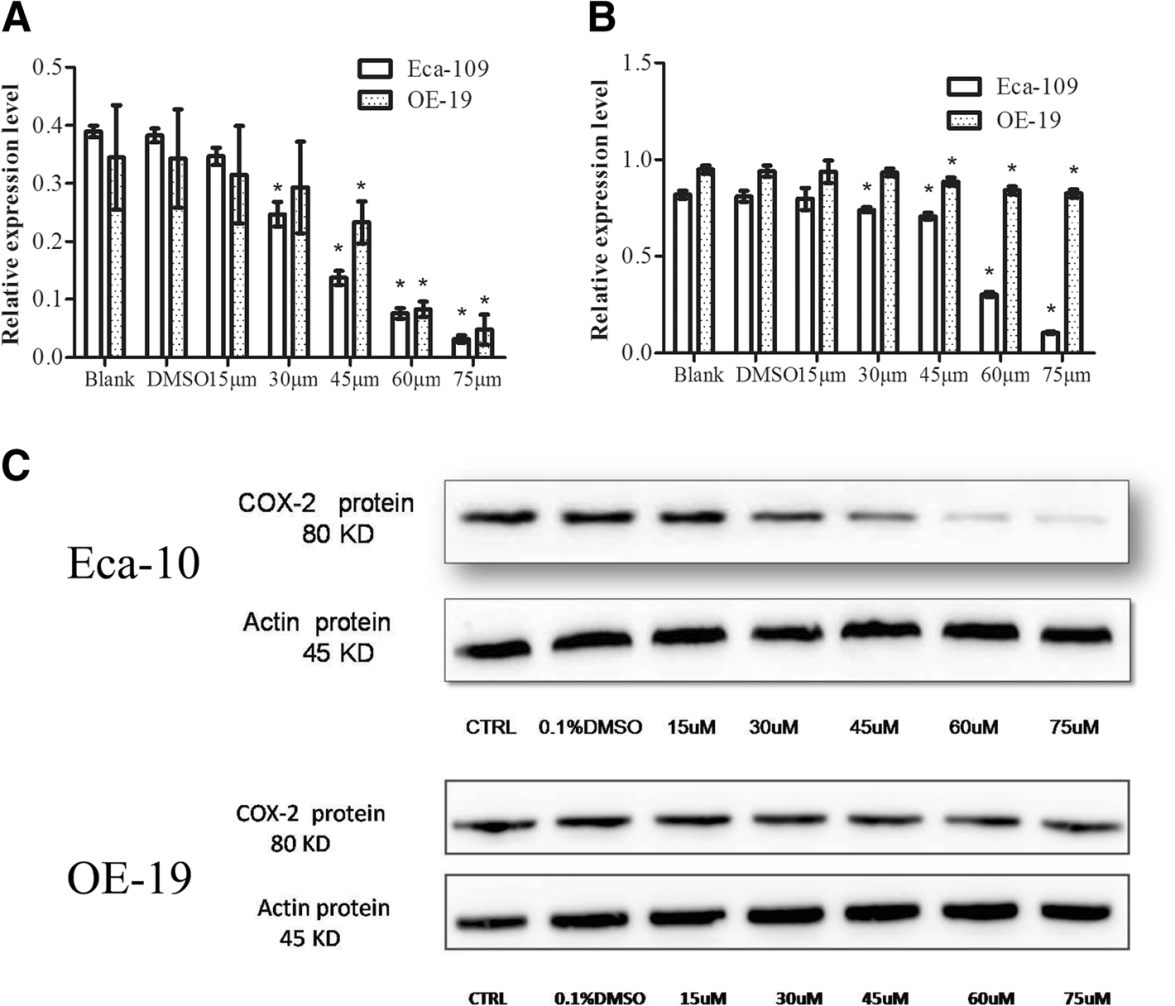
Effect of simvastatin treatment on COX-2 expression in OE-19 and Eca-109 cells by study group. **a** OE-19 and Eca-109 cells were treated with 0.1% DMSO or different concentrations (15 μM, 30 μM, 45 μM, 60 μM and 75 μM) of simvastatin for 24 h. Total RNA was extracted for determination of mRNA levels of COX-2 with GAPHD as control. **b** OE-19 and Eca-109 cells were treated as indicated for 24 h. Total protein was extracted for immuoblotting of COX-2, using β-actin as loading control. The relative amounts of each protein were quantified by densitometry as ratios to β-actin. **c** Representative Western blot results for COX-2 and β-actin expression. * indicates significant difference from DMSO group (*P* < 0.05). COX-2, Cyclooxygenase-2; *Eca-109, Esophageal squamous cell carcinoma; OE-19, esophageal adenocarcinoma*; DMSO, *dimethylsulfoxide*; TBA, *thiobarbituric acid*

Simvastatin (> 15 μM) suppresses both cell lines’ COX-2 mRNA and protein expression in a dose-dependent way (*P* < 0.05) (Fig. 3a-c).

### Simvastatin suppressed the level of PGE_2_

**Fig. 4:**
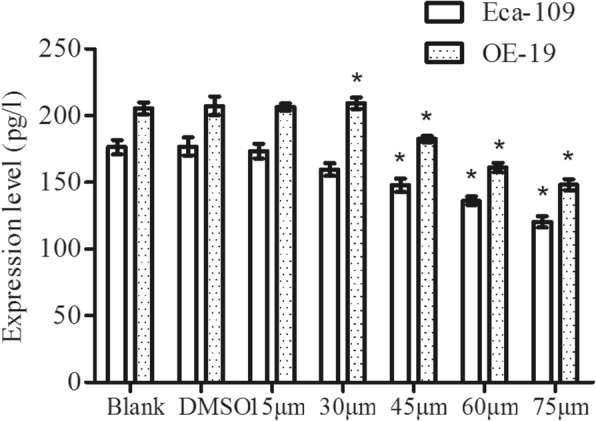
Results of ELISA for expression of PGE_2_ in OE-19 and Eca-109 cells by study group of Simvastatin. * indicates significant difference from DMSO group (*P* < 0.05). *ELISA, Enzyme linked immunosorbent assay; Eca-109, Esophageal squamous cell carcinoma; OE-19, esophageal adenocarcinoma*

Simvastatin had a dose-dependent suppression effect on both OE-19 and Eca-109 cells. Further statistical analysis revealed that simvastatin had the most notable effect from a dose of 30 μM and 45 μM respectively (*p* < 0.05) (Fig. 4).

## Discussion

As early as 1996, researchers proposed that most cholesterol-lowering drugs were associated with cancer in rodents. Patients to whom these drugs (including statins) are prescribed are exposed throughout many years to doses approaching those shown to be carcinogenic in animals. However, there was uncertainty in extrapolating results of carcinogenicity studies from rodents to humans, the implication of these findings dismatched that of meta-analyses of clinical trials in humans. Heart Protection Study [11] involved 20,536 high-risk patients with coronary heart disease, and found that there were no significant adverse effects on cancer incidence between patients with simvastatin (40 mg once daily) for 5 years and control group. A study [12] aimed to investigate the mortality and incidence of cancer during 10-year follow-up showed that simvastatin (20 mg or 40 mg once daily) produced lasting benefits of survival and may not increase the risk of cancer.

In our study, simvastatin, but not pravastatin, inhibited the proliferation of OE-19 and Eca-109 cells in a dose- and time-dependent manner, accompanying with the increasing of MDA. Further more, simvastatin suppressed the expression of COX-2 and PGE_2_ in both cell types in a dose-dependent manner.

Although inhibition of HMG-CoA reductase,which leading to reduced cholesterol biosynthesis, is the most widely appreciated biological action of statins, there are several other pharmacological effects. Statins had been shown to reduce the risk of many cancers [4, 5], however, the underlying mechanism has not been well defined. Therefore, it is important to elucidate the effects of statins on the cell proliferation in different cancers. In the present study, lipophilic simvastatin at the concentration of 30 μM significantly inhibited the proliferation of both Eca-109 and OE-19 cells in a time-dependent manner, while similar effect was not observed with hydrophilic pravastatin. Similarly, lipophilic, but bot hydrophilic stains, have been shown to reduce proliferation in colorectal cancer, breast cancer, thyroid cancer cells in vitro [13]. Whether solubility of stains affects the proliferation of malignant cells remains unclear. Most of these studies were conducted *in vitro*, which can not provide the chance to observe the intermediate products after complex metabolism and interactions among different cell types. Moreover, tumor cells themself may have various metabolic abnormalities. Ogunwobi et al. [14] found that all the lipophilic simvastatin, lovastatin, and hydrophilic pravastatin inhibited Ras farnesylation and proliferation of OE33 and BIC-1 cells. Cells derived from different tissues may react with the solubility of stains in different ways. Menter et al. assessed the antiproliferative activity of simvastatin and pravastatin to cancer cells derived from colon, pancreatic, breast, liver and other tissues, as well as normal liver tissue cells. They found that simvastatin inhibited the proliferation of all the cell lines, however, pravastatin only acted on normal liver cells, which might be related to organic anion transporter peptide (OATP1B1) on the surface of liver cells [15]. OATP1B1 is present in normal liver cells, but not in colorectal and liver cancer cells [16, 17]. Turther detection of whether OATP1B1 exists on EC cells may contribute to clarify the useless of hydrophilic stains to the proliferation of EC cells.

Lipid peroxidation is one of oxidative stress reaction which could seriously injure intracellular proteins, lipids and nucleic acids and induce cell death. The level of MDA, a marker of lipid peroxidation, was known to correlate with the rate of lipid peroxidation [18, 19]. In our study, contrary to pravastatin, simvastatin up-regulated the level of MDA apparently. However, Sathyapalan et al. [20] reported that both simvastatin and atorvastatin significantly decreased levels of MDA and hsCRP levels in type 2 diabetic patients. Atorvastatin has also been shown to significantly reduce MDA levels and oxidative stress in diabetic rats, which appears to prevent cardiovascular events. We tend to attribute this phenomenon to reduction in cholestrin synthesis [21]. Moreover, atorvastatin significantly inhibited the expression of TNF-α and MCP-1 induced by OX-LDL in RAW264.7 mouse macrophage cell line [22], suggesting that statins can antagonize inflammation, independent of their lipid-lowering effects.

Many studies have shown that COX-2 had been implicated in the induction of ESCC [23–25]. However, selective COX-2 inhibitors did not enhance the antiproliferative effect in ESCC, gastrointestinal and cardiovascular side effects for long-term use restricted their clinical application [26, 27]. We have shown that simvastatin suppressed the expression of COX-2 and PGE2 in both OE-19 and Eca-109 cells in a dose-dependent manner without the side effects of gastrointestinal and cardiovascular. Accordingly, stains may be potentially promising strategy for the chemoprevention and management of EC.

Unlike our study in vitro, epidemiological studies [9] found that lipophilic and hydrophilic statins had a negative correlation with EC (OR = 0.86, 95%CI = 0.75 to 0.98 and OR = 0.71, 95%CI = 0.51 to 0.98, respectively), indicating hydrophilic statins had stronger antitumor effects. In order to elucidate the underlying mechanisms involved in the suppression of esophageal cancer by statins, kinds of stains with different solubility need to be tested on a variety of EC cell lines.

Since neovascularisation is important for the growth of primary tumors and metastasis, the effects of statins on angiogenesis have been assessed. It was interesting that low concentrations of statins enhanced endothelial cell proliferation, whereas relatively high doses inhibited angiogenesis by decreasing the levels of vascular endothelial growth factor (VEGF) by unknown mechanism [28]. In our study, we have shown that simvastatin significantly inhibited serum-induced proliferation in both OE-19 and Eca-109 cells at concentrations of 30 μM and above. This concentration is comparable to those used in other in vitro studies [2], higher than the dosage to treat hyperlipidemia [29, 30] (0.1–0.5 μM). Additionally, in another experiment, investigators [31] found that lovastatin inhibits tumor grouth at a high dosage, however it promotes tumor growth at a low dosage. Whether this duality effect is related to different levels of VEGF is still uncertain.

Moreover, along with the rapid development of statins applications, security draws lots of attention. In a meta-analysis of 90 studies [32], statins were associated with lower risks of dementia and cognitive impairment, venous thrombo-embolism, fractures and pneumonia. Statin use was not related to any increased risk of depression, renal disorders or arthritis. There was evidence of an increased risk of myopathy, diabetes mellitus, and raised liver enzymes. Intensive-dose therapy with atorvastatin or simvastatin (80 mg/day) was associated with an increased risk for abnormalities on liver function testing [33]. On the other hand, a meta-analysis of de Denus et al. [34] found that pravastatin, lovastatin, and simvastatin at low-to-moderate doses are not associated with a significant risk of liver function test abnormalities. In our study, simvastatin inhibited the proliferation of EC in a dose-dependent manner. Simvastatin is metabolized by CYP3A4, drug induced liver injury is associated with increased drug dosage [35]. Moderate-dose statin therapy (simvastatin of 20–40 mg once daily in human body, equal to 48–96 μM) may be the most appropriate choice for achieving cancer risk reduction in the majority of individuals.

The metabolism and pharmacokinetics of the different statins may relate to potential anticancer actions. The more lipophilic statins, such as lovastatin, are typically metabolized to moieties with HMG-CoA reductase activity of their own, and thus serum levels of the parent compound may not reflect total bioactivity [31]. This makes correlation between different studies with different assay techniques difficult. Compared to traditional chemotherapy drug, the toxicity and pharmacokinetics of stains are still awaiting further large-scale clinical trials.

## Conclusion

Our study suggested that lipophilic simvastatin but not hydrophilic pravastatin have a significant inhibition effect on the proliferation of Eca-109 and OE-19 cells, accompanying with the down-regulation of COX-2 and PGE2, which was independent of lipid-lowering effects. Simvastatin may have a role in the prevention or treatment of EC.

## Abbreviations

COX-2: Cyclooxygenase-2
EAC: Esophageal adenocarcinoma
EC: Esophageal cancer
ESCC: Esophageal squamous cell carcinoma
HMG- CoA: hydroxymethylglutaryl coenzymeA
MDA: Malondialdehyde
PGE_2_: Prostaglandin E_2_
RT-PCR: Reverse transcriptase-polymerase chain reaction
TBA: Thiobarbituric acid
VEGF: Vascular endothelial growth factor
